# First-in-human Phase 1 open label study of the BET inhibitor ODM-207 in patients with selected solid tumours

**DOI:** 10.1038/s41416-020-01077-z

**Published:** 2020-09-29

**Authors:** Malaka Ameratunga, Irene Braña, Petri Bono, Sophie Postel-Vinay, Ruth Plummer, John Aspegren, Timo Korjamo, Amir Snapir, Johann S de Bono

**Affiliations:** 1 The Institute of Cancer Research and Royal Marsden, London, UK; 2 Vall d’Hebrón Institut d’Oncologia, Barcelona, Spain; 3 Helsinki University Hospital Comprehensive Cancer Center, Helsinki, Finland; 4 University of Helsinki, Helsinki, Finland; 5 Drug Development Department, DITEP, Gustave Roussy, Villejuif, France; 6 Translational and Clinical Research Institute, Newcastle University and Newcastle Hospitals NHS Foundation Trust, Newcastle upon Tyne, UK; 7 Orion Corporation Orion Pharma, Espoo, Finland; 8 Present Address: Monash University, Melbourne, Australia; 9 Present Address: Terveystalo Finland and University of Helsinki, Helsinki, Finland; 10 Present Address: PCI Biotech, Oslo, Norway

**Keywords:** Drug development, Pharmaceutics, Prostate cancer, Breast cancer, Melanoma

## Abstract

**Background:**

Bromodomain and extra-terminal domain (BET) proteins are reported to be epigenetic anti-cancer drug targets. This first-in-human study evaluated the safety, pharmacokinetics and preliminary anti-tumour activity of the BET inhibitor ODM-207 in patients with selected solid tumours.

**Methods:**

This was an open-label Phase 1 study comprised of a dose escalation part, and evaluation of the effect of food on pharmacokinetics. ODM-207 was administered orally once daily. The dose escalation part was initiated with a dose titration in the initial cohort, followed by a 3 + 3 design.

**Results:**

Thirty-five patients were treated with ODM-207, of whom 12 (34%) had castrate-resistant prostate cancer. One dose-limiting toxicity of intolerable fatigue was observed. The highest studied dose achieved was 2 mg/kg due to cumulative toxicity observed beyond the dose-limiting toxicity (DLT) treatment window. Common AEs included thrombocytopenia, asthenia, nausea, anorexia, diarrhoea, fatigue, and vomiting. Platelet count decreased proportionally to exposure with rapid recovery upon treatment discontinuation. No partial or complete responses were observed.

**Conclusions:**

ODM-207 shows increasing exposure in dose escalation and was safe at doses up to 2 mg/kg but had a narrow therapeutic window.

**Clinical trial registration:**

The clinical trial registration number is NCT03035591.

## Background

Acetylation of lysine residues on histone tails is associated with open chromatin and transcriptional activation.^1^ Bromodomains are a highly evolutionarily conserved family of proteins responsible for binding acetylated lysine residues at the amino-terminal tails of histones.^2^ The bromodomain and extra-terminal (BET) family (BRD2, BRD3, BRD4 and BRDT) contain two tandem bromodomains which bind acetylated lysine residues, facilitating recruitment of transcriptional proteins to chromatin.^3^ BRD4 localises to promoter and enhancer regions of chromatin, and functions to regulate RNA-pol II-mediated elongation and transcription through interactions with the Mediator complex and pTEFb.^4,5^ In many tumour types, the efficacy of BET inhibitors has been attributed to the transcriptional suppression of genes such as *MYC*.^6^ More significantly, evidence of the oncogenic nature of BRD4 is apparent in the rare and aggressive tumour known as nut midline carcinoma (NMC), which occurs as the result of a chromosomal translocation with a resultant fusion protein of BRD4 with nuclear protein in testis (NUT).^7^ Although less common, the BRD3-NUT fusion protein may confer a better prognosis whilst still potentially responding to therapeutic inhibition with BET inhibitors.^8,9^ Importantly, there is evidence that preclinical activity of various BET inhibitors in various tumour types can be both MYC dependent^6^ and MYC independent.^10^ Consequently, the BET family of proteins are a promising candidate for anti-cancer therapy.

Several small molecule BET inhibitors, predominantly structurally related to JQ1, are in clinical development and have shown some preliminary clinical activity in NMC and lymphoma.^11–14^ These studies showed BET inhibition strongly correlated with decreasing platelet count.^13,14^ ODM-207, a novel small molecule that is structurally unrelated to JQ1, is a highly potent pan-BET inhibitor that has shown preclinical evidence of tumour growth inhibition in breast cancer and in leukaemia and prostate cancer xenograft models.^15–18^ ODM-207 has shown minimal cross-reactivity with non-BET family bromodomains.

Here, we describe the first-in-human Phase 1 study designed to investigate the safety, pharmacokinetics and maximum tolerated dose (MTD) of ODM-207 in patients with advanced malignancies. Platelet count was chosen as a pharmacodynamic biomarker based on the observed robust correlation between BET inhibitor exposure and thrombocytopaenia in preclinical and clinical studies.^13,14^

## Methods

### Study design

This was a Phase 1–2, multicentre, non-randomised, open-label, dose-escalation study of ODM-207 (NCT03035591, Orion Corporation) in patients with selected solid tumours. The primary objectives were to determine the safety and tolerability, dose-limiting toxicities (DLT), and maximum tolerated dose (MTD) of ODM-207. The secondary objectives of the study were to characterise the pharmacokinetics of ODM-207 and its main metabolite, to evaluate the effects of food on bioavailability and to generate preliminary evidence of target inhibition and anti-tumour activity.

The study design contained two parts: (1) dose escalation in patients with advanced solid tumours with an innovative single dose accelerated titration procedure in the first cohort (1A), (2) evaluation of food effect on bioavailability (1B). The single-dose accelerated titration procedure in the first cohort involved comparison of measured pharmacokinetic parameters in the first cohort of patients against preclinical models to evaluate whether exposure was within the target range. If the measured exposure was outside the target range (below or above), the protocol allowed for dose adjustment. Dose escalation was performed using a traditional 3 + 3 schedule. The food effect cohort involved a single fasted dose (overnight fasting, food allowed 4-h after dosing); after a washout period ODM-207 was given after a light breakfast (200–300 kcal of which 50–100 kcal from fat). The relevant national authorities provided regulatory approval and local institutional review boards approved the protocol. All study procedures were conducted in accordance with the Declaration of Helsinki and good clinical practice. All patients provided written informed consent.

### Patients and drug administration

Patients in the dose escalation part received oral ODM-207 once daily in a 28-day cycle at a starting dose of a 50 mg tablet daily under fed conditions. The starting dose was chosen as it was reasonably expected to be both pharmacologically active and safe and tolerable. The target exposure for the starting dose was estimated from exposures observed in pre-clinical anti-tumour activity and safety studies. The estimated human dose required was predicted by both human equivalent dose calculations and physiologically-based pharmacokinetic modelling. A single dose titration procedure was performed in the first cohort of patients to confirm that exposure of ODM-207 in humans corresponded with the pre-defined target exposure based on non-clinical data. The starting dose provided plasma exposure that was in the target range. Therefore, no further dose titration in the first cohort was pursued (Fig. S1). Treatment was taken in the ambulatory setting, except for hospital admissions required for pre-specified study-related procedures.

Eligible patients had histologically confirmed NMC, high-grade serous ovarian cancer, HER2 negative breast cancer, castrate-resistant prostate cancer, small cell lung cancer, non-small cell lung cancer, melanoma, non-Hodgkin lymphoma, sarcoma or any other tumour predicted to have a significantly higher likelihood of response to ODM-207 (such as *MYC* amplified tumours), with no effective standard therapy available. Patients had to have ECOG performance status ≤ 1, adequate haematopoietic, renal, hepatic and coagulation function, life expectancy >12 weeks and needed a washout period from prior anti-cancer therapy.

### Procedures and safety assessments

Safety was assessed at baseline, continuously during treatment and for 28 days following cessation of ODM-207. The National Cancer Institute Common Terminology Criteria for Adverse Events (version 4.03) were used to grade treatment-emergent adverse events (AEs).^19^ DLTs, defined as AEs related to ODM-207 that occurred within the first 28 days (cycle 1), included grade 4 neutropaenia lasting more than seven days, febrile neutropaenia, grade 3 thrombocytopaenia with grade 3 bleeding or grade 4 thrombocytopaenia lasting more than three days, treatment interruption of 14 days, persistent grade 3 gastrointestinal toxicity despite optimal medical intervention, grade 3 liver function abnormalities lasting more than seven days. The MTD was defined at the highest dose at which <33% of patients experienced DLTs during cycle 1 within a cohort. Response was assessed using RECIST 1.1 for patients with advanced solid tumours,^20^ Prostate Cancer Working Group 3 criteria (PCWG3) for castrate-resistant prostate cancer^21^ and Lugano criteria for non-Hodgkin lymphoma.^22^ Blood samples were taken at nine time points on initial dosing, after two weeks of dosing and before dosing in all follow up visits to establish the pharmacokinetic profile (maximum concentration, maximum time, area under the plasma concentration-time curve, volume of distribution, clearance and half-life, and minimum concentration at long term steady state) of ODM-207.

### Statistical analyses

Safety analyses included all patients who received any doses of treatment. Efficacy evaluation was performed on all patients who received two or more cycles of treatment (≥8 weeks). Data points were excluded from relevant pharmacokinetic and platelet count analyses if samples were taken when the patient was on treatment interruption or within 3 days after resumption of treatment. Three levels of exposure (low, mid, high) were defined based on pre-clinical pharmacological in-house studies about ODM-207 and observed effects of ODM-207 on count of platelets. Pre-dose total exposure of <3500 ng/mL was not expected to have significant BET inhibition effect and a level of ≥5000 ng/mL was expected to confer BET inhibition.

## Results

Between 22 December 2016 and 10 May 2019, 36 patients were enrolled, and 35 patients were treated at doses ranging from 50 mg to 205 mg corresponding with a range of 0.6–2.0 mg/kg. After a dose-limiting toxicity of fatigue was observed in one subject in cohort 1A-2 (100 mg), a decision was made to change the dosing regimen to weight-based dosing due to a wide range of exposure and assumed narrow therapeutic range. Dose escalation continued with mg/kg-based dosing up to the level of 2 mg/kg. This dose level was determined to be the MTD based upon observed adverse events, although further DLTs were not observed. The demographic information is shown in Table 1. Of the 35 treated patients, 12 (34%) had castrate-resistant prostate cancer (CRPC), 5 (14%) had melanoma, 4 (11%) had NMC, 4 (11%) had oestrogen receptor positive breast cancer (ER + BC) and 10 (29%) had other tumour types. Median age was 60 years (range 17–78 years). Treatment discontinuation occurred due to disease progression in 28 (80%) patients, due to AEs in 5 (14%) patients and 2 (6%) for other reasons.

**Table 1:** Patient demographics.

Characteristic	1A (dose escalation) *N* (%)	1B (dosing adjustment) *N* (%)	Total *N* (%)
Number of patients	21 (60)	14 (40)	35 (100)
Age, years, median (range)	63 (31–78)	54 (17–74)	60 (17–78)
Sex			
Female	9 (43)	5 (36)	14 (40)
Male	12 (57)	9 (64)	21 (60)
Cancer type			
CRPC	8 (38)	4 (29)	12 (34)
Melanoma	2 (10)	3 (21)	5 (14)
NMC	0 (0)	4 (29)	4 (11)
ER + BC	4 (19)	0 (0)	4 (11)
Other	7 (33)	3 (21)	10 (29)
ECOG PS			
0	10 (48)	3 (21)	13 (37)
1	11 (52)	11 (79)	22 (63)
≥3 Prior lines of antineoplastic treatment	19 (90)	10 (71)	29 (83)

*CRPC* castration resistant prostate cancer, *NMC NUT* midline carcinoma, *ER* *+* *BC* oestrogen receptor positive breast cancer, *PS* performance status.

### Dose escalation

O 36 patients enrolled in the study, 35 were evaluable for safety (one patient did not receive ODM-207 due to rapidly deteriorating performance status). Based on the pharmacokinetic results from single-dose titration in the first cohort of patients no adjustment was required for the starting dose. One patient receiving a dose of 100 mg (1.9 mg/kg) experienced a DLT of intolerable grade 3 fatigue which was attributable to ODM-207 and this cohort (1A-2) was expanded. Although dose escalation continued up to the dose of 2 mg/kg without further dose-limiting toxicities, the safety monitoring board determined this to be the maximum tolerated dose due to ongoing intolerable fatigue and nausea, necessitating dose reduction and/or treatment discontinuation beyond the DLT period at this dose level. At the dose level of 2 mg/kg, five patients out of six had AEs resulting in dosing interruption and of these two patients required dose reductions and one discontinued the study. The dose-limiting toxicities and grade 3 adverse events are shown in Table 2.

**Table 2:** DLTs and grade 3 adverse events.

	1A-1 0.6 mg/kg^a^ (*n* = 3)	1A-2 1.3 mg/kg^a^ (*n* = 7)	1A-3 1.5 mg/kg (*n* = 5)	1A-4 2 mg/kg (*n* = 6)	1B-1 1.1 mg/kg (*n* = 10)	1B-2 1.4 mg/kg (*n* = 4)	Total (*n* = 35)
	*n* (%)	*n* (%)	*n* (%)	*n* (%)	*n* (%)	*n* (%)	*n* (%)
SAEs		2 (29)	2 (40)	3 (50)	3 (30)	4 (100)	14 (40)
DLTs		1 (14)					1 (2.9)
Related SAEs				2 (33)		1 (25)	3 (8.6)
Grade ≥ 3 AEs		3 (43)	2 (40)	4 (67)	6 (60)	4 (100)	19 (54)
AEs leading to study discontinuation	1 (33)		2 (40)	1 (17)	3 (30)	2 (50)	9 (26)
AEs leading to dose reduction				2 (33)	1 (10)	1 (25)	4 (11)
AEs leading to dosing interruption	2 (67)	4 (57)	2 (40)	5 (83)	4 (40)	2 (50)	19 (54)

^a^Cohorts 1A-1 and 1A-2 were given the doses 50 and 100 mg, respectively, presented in Table 2 as cohort average in mg/kg. The range of doses in cohort 1A-1 was 0.1–0.7 and in 1A-2 was 0.9–1.9 mg/kg.

### Safety

Overall 31 (89%) patients experienced a treatment-related AE. Treatment emergent related AEs, which occurred in a frequency of >10% of patients and/or of grade 3 severity are shown in Table 3. There were five deaths on study, all of which were considered not related to ODM-207 (one patient died of bowel obstruction, two of respiratory distress and two of disease progression). Common AEs included nausea (66%), thrombocytopaenia (51% of patients reported any grade), anorexia (49%), fatigue (43%), diarrhoea (40%), vomiting (40%), headache (37%) and weight loss (23%). Most common treatment-related grade ≥3 adverse events included thrombocytopaenia (14.3%), nausea (8.6%) and fatigue (5.7%).

**Table 3:** Related AEs.

	Total (*n* = 35)			
	GRADE 1	GRADE 2	GRADE 3	GRADE 4
System	*n* (%)	*n* (%)	*n* (%)	*n* (%)
Haematologic				
Thrombocytopaenia	15 (43)	8 (23)	4 (11)	1 (2.9)
Gastrointestinal				
Diarrhoea	9 (26)	5 (14)		
Nausea	13 (37)	4 (11)	3 (8.6)	
Vomiting	7 (20)	3 (8.6)		
General				
Asthenia	3 (8.6)	3 (8.6)		
Fatigue	9 (26)	6 (17)	2 (5.7)	
Decreased appetite	12 (34)	8 (23)		
Weight loss	4 (11)	1 (2.9)	1 (2.9)	
Nervous system				
Dysgeusia	4 (11)	3 (8.6)		
Headache	7 (20)	3 (8.6)		

### Pharmacokinetics

**Fig. 1:**
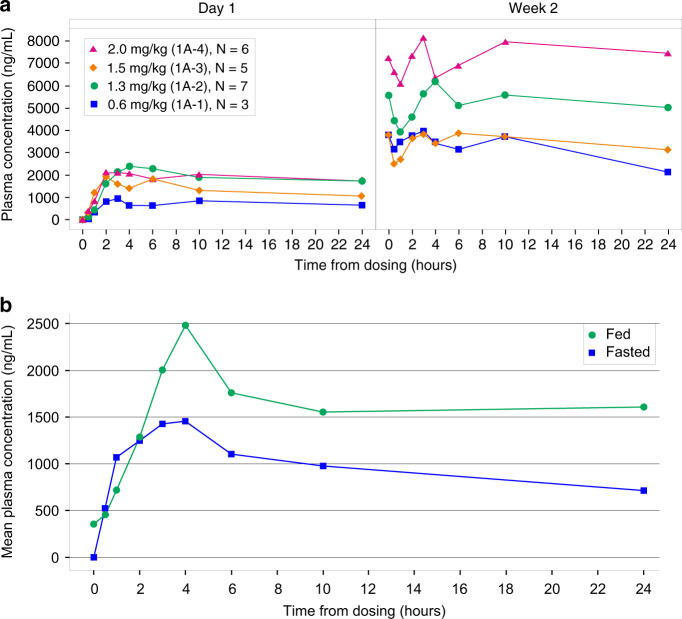
Pharmacokinetics of ODM-207. Cohort mean concentrations in fed-state on day 1 and day 15 (**a**), and in fed/fasted state in one cohort (**b**). **a** Cohorts 1A-1 0.6 mg/kg (blue square), Cohort 1A-2 1.3 mg/kg (green circle), Cohort 1A-3 1.5 mg/kg (orange diamond), and Cohort 1A-4 2.0 mg/kg (pink triangle). **b** Fasted state (blue square) and fed state (green circle).

Analysis of plasma pharmacokinetics showed slow absorption and elimination of ODM-207 with a median time to peak concentration between 2 and 6 h. Mean ODM-207 AUC_(0-t)_ increased between day 1 and day 15 of dosing by 3.4-fold (*n* = 28, 95% CI 2.9, 3.8). Cohort mean total plasma concentrations on day 1 and day 15 (steady state) are presented in Fig. 1a. Plasma exposure to ODM-207 (AUC_0-24_) under fed condition was higher 1.5-fold than under fasting condition (Fig. 1b). The main metabolite is a demethylation product of ODM-207. The metabolite/parent ratio based in AUC_(0-t)_ was 0.1–0.2 on day 1 and 0.1–0.5 on day 15.

### Pharmacodynamics

**Fig. 2:**
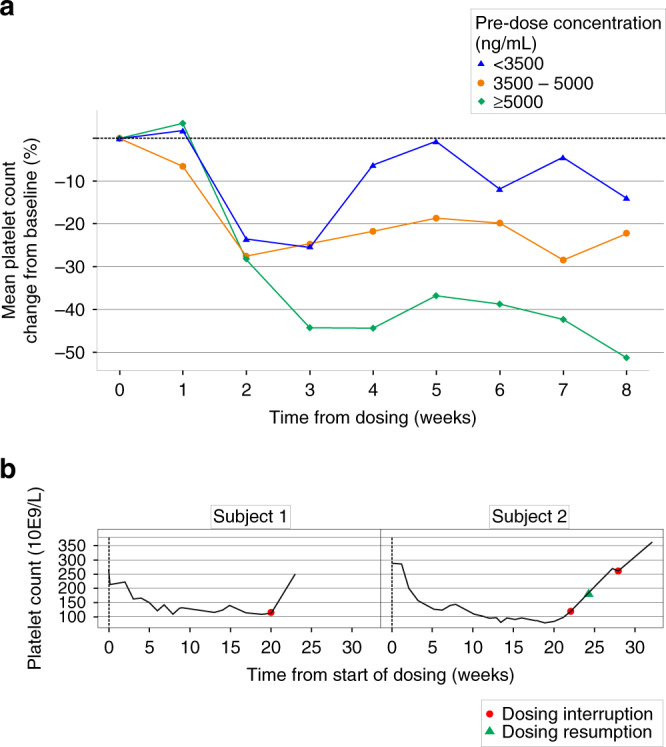
Platelet count change over time. Mean platelet change from baseline (%) by pre-dose ODM-207 exposure (**a**) and examples of recovery of platelets upon dosing interruption (**b**). **a** Pre-dose concentration (ng/mL) <3500 (blue triangle), 3500–5000 (orange circle), and ≥5000 (green diamond). **b** Dosing interruption (orange circle) and resumption (green triangle).

As was shown with other BET inhibitors,^13,14,23^ a strong correlation was observed between ODM-207 plasma exposure and changes in platelet count over time. Prompt recovery of platelet count upon treatment interruption was observed (Fig. 2).

### Anti-tumour activity

**Fig. 3:**
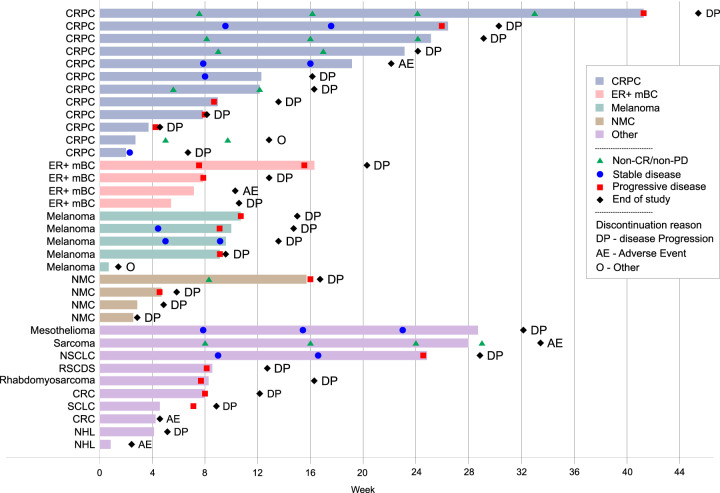
Duration of treatment and reason for discontinuation by cancer type. CRPC castration-resistant prostate cancer, ER + mBC oestrogen receptor positive metastatic breast cancer, NMC NUT midline carcinoma, NSCLC non-small cell lung cancer, RSCDS round small cells desmoplastic sarcoma, CRC colorectal carcinoma, SCLC small cell lung cancer, NHL non-Hodgkin’s lymphoma, SD stable disease, PD progressive disease, CR complete response, EOS end of study.

Of the 27 patients with RECIST tumour response data, 21 were evaluable (completing ≥ 8 weeks of treatment). None had a partial or complete response (PR, CR). Six patients (29%) had stable disease (SD), 9 (43%) had progressive disease (PD), and 6 (29%) had non-CR/non-PD (non-measurable disease) as their best response. Eight patients (38%) had either SD or non-CR/non-PD at 16 weeks. The median (range) duration of SD was 16.3 (8.0–23.0) weeks. One out of four patients with NMC had SD as their best response, the other patients had clinical progression prior to their first disease assessment. The median progression-free survival was 2.2 months (range 0.5–9.7 months). Duration on treatment, ongoing RECIST response and reason for study discontinuation by patient’s tumour type are summarised in Fig. 3. There was no correlation between progression-free survival and increased exposure. None of the prostate cancer patients showed decline in PSA below baseline level.

## Discussion

The aim of this first-in-human Phase 1 study was to assess the safety and tolerability of the BET inhibitor ODM-207. The main dose-limiting adverse events were nausea and fatigue and the maximal tolerated dose of 2 mg/kg was identified. Other significant observed toxicities included asthenia and anorexia. Consistent with other studies of BET inhibitors, reversible thrombocytopaenia was observed,^12,24^ which further validates platelet count as a potential pharmacodynamic marker of on-target BET inhibition. We could not validate ODM-207’s therapeutic efficacy in NMC because the enrolled patients either progressed within days of enrolment (3 out of 4) or had very low ODM-207 plasma exposure (1 patient). None of these patients developed thrombocytopaenia. Thus, the lack of activity in these patients reflects inadequate exposure, which when coupled with an aggressive underlying disease, resulted in rapid disease progression.

A unique aspect of this clinical trial was the novel dose titration methodology utilised in the first cohort of patients. Plasma pharmacokinetic parameters were sampled after single dosing and were evaluated against preclinical models of exposure, providing confidence regarding the exposure and pharmacokinetic profiles in humans and mitigating the risk of inaccurate translations from models to humans and the risk for too low or too high dose in the first cohort of the first-in-human study. This methodology is particularly useful for first-in-human studies in which preclinical confidence in bioavailability is limited due to significant variability between models, or as in this case, where a narrow therapeutic window is expected.

In this study, modelling was performed over a short duration of 7 days, and also confirmed that exposure in the first cohort was at target level based on non-clinical data, demonstrating that rapid pharmacokinetic assessment is possible within a clinical trial setting whilst minimising the risk of inadequate exposure to this cohort of patients with advanced cancer. Although not demonstrated within this trial, this design could theoretically also reduce the number of dose escalation steps if dose titration was required, saving unnecessary burden from patients, either treated with a too low or high dose. Such a procedure can also significantly decrease development time and use of resources. In addition, the food-effect cohort included within this study, robustly demonstrated the association of the fed state with increased exposure. Overall at the higher dose levels, exposure was within the range expected for clinical activity. Target inhibition was confirmed with the consistent exposure-dependent decrease in platelet count observed across the study.

Multiple first-in-human studies of BET inhibitors have now been published, with the majority of compounds being analogues of JQ1, the first identified BET inhibitor.^3^ The chemical composition of ODM-207 is distinct from JQ1 analogues, but notably had overlapping profile of adverse events with other compounds.^12,24^ Consistently with other BET inhibitors, treatment-related adverse events limited dose escalation with most commonly observed AEs being thrombocytopenia, nausea, fatigue, decreased appetite, and diarrhoea.

Eight (38% of 21 evaluable patients) had stable disease or non-CR/non-PD for 16 weeks or more. The lack of significant responses is consistent with other studies of BET inhibitors, none of which have observed significant responses in solid tumours.^11,12,24^ The results of this study, when contextualised with the results of other Phase 1 studies of BET inhibitors in solid tumours, convey evidence of no strong signal of efficacy as a monotherapy in tumour types other than NMC. Mechanistically, the BET family of proteins control a diverse array of cellular processes^6^ and more preclinical work needs to be done prior to elucidation of the appropriate clinical context wherein BET inhibitors may be best utilised.^25^ The results of this trial herein, and emerging data from other Phase 1 studies of BET inhibitors in solid tumours, suggest that achieving a therapeutic window where an anti-tumour effect can be achieved without undue adverse events due to on-target effects may be challenging with this class of agents, also making drug combinations particularly difficult. Nevertheless, the diverse transcriptional networks impacted by BET inhibition have raised multiple combination opportunities, the goal being more favourable risk/benefit ratios. Of particular interest are combinations with immune checkpoint inhibitors,^26,27^ PARP inhibitors,^28,29^ CDK4/6 inhibitors or similar.^30,31^

In conclusion, evaluation of the BET inhibitor ODM-207 in this first-in-human study demonstrated dose proportional exposure with reversible thrombocytopaenia and in higher dose levels, fatigue and nausea, consistent with other studies of BET inhibitors. Strong signal for anti-tumour activity was not observed in patients with the selected advanced solid tumours.

## Supplementary information

Supplemental Material File #1

## Data Availability

The datasets generated and/or analysed during the current study are not publicly available due proprietary restrictions but are available from the corresponding author on reasonable request.
