# Supplementary material for: First-in-human Phase 1 open label study of the BET inhibitor ODM-207 in patients with selected solid tumours
Source: Br J Cancer. 2020 Sep 29;123(12):1730–6. doi: 10.1038/s41416-020-01077-z (PMC7722752; doi:10.1038/s41416-020-01077-z)
Supplement: Supplementary file 1 — Supplemental Material File #1 [file 41416_2020_1077_MOESM1_ESM.pdf]

Supplemental information of first cohort and run-in single-dose titration procedure

If the observed plasma exposure after the single dose is outside the desired range, the SMB will consider the following and will make appropriate decisions (Figure S1):

1. Is dose adjustment needed?
2. If dose adjustment is needed, is it likely that an adjusted dose will allow the reaching of the desired exposure?
3. If the dose can be adjusted to allow the desired exposure, what should be the new dose level? The adjustment will be based on the patient's PK data after single dose administration.
4. After determining the adjusted dose, a decision needs to be made on whether the patient can continue treatment with repeated dosing or is there a need first to re-evaluate single dose PK data after dose adjustment before repeated dosing begins.

Figure S1. Decision flowchart on escalation of dose in the first cohort

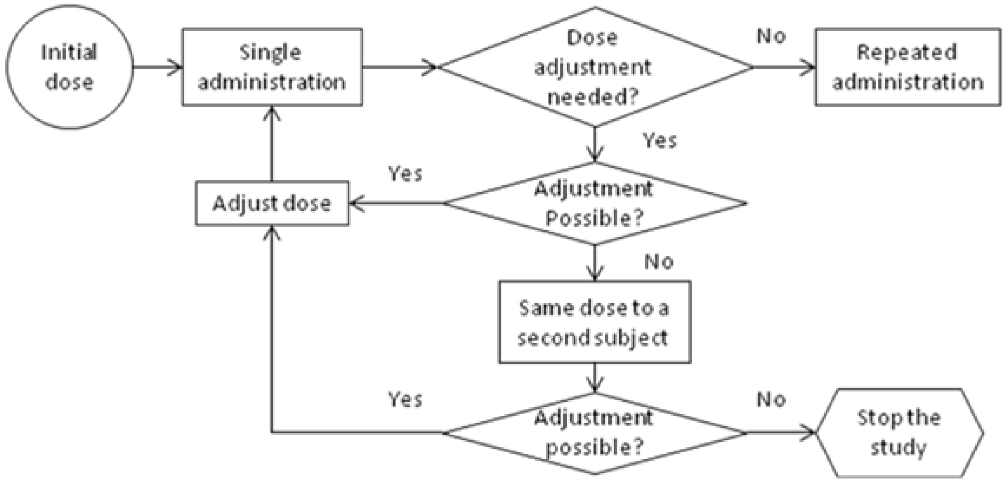


If dose adjustment is recommended for a specific patient, a single dose of ODM-207 will be administered to that patient at a new dose level, 1-2 weeks after the first dose. Blood will be collected before and up to 24 h after the administration of the single dose of ODM-207 and PK data will be evaluated.
